# Advances in Engineered Polymer Nanoparticle Tracking Platforms towards Cancer Immunotherapy—Current Status and Future Perspectives

**DOI:** 10.3390/vaccines9080935

**Published:** 2021-08-23

**Authors:** Ramar Thangam, Kapil D. Patel, Heemin Kang, Ramasamy Paulmurugan

**Affiliations:** 1 Department of Materials Science and Engineering, Korea University, Seoul 02841, Korea; dynamic2020@korea.ac.kr (K.D.P.); heeminkang@korea.ac.kr (H.K.); 2 Institute for High Technology Materials and Devices, Korea University, Seoul 02841, Korea; 3 Department of Biomicrosystem Technology, Korea University, Seoul 02841, Korea; 4 Department of Radiology, Molecular Imaging Program at Stanford, School of Medicine, Stanford University, Palo Alto, CA 94304, USA; 5 Department of Radiology, Canary Center at Stanford for Cancer Early Detection, School of Medicine, Stanford University, Palo Alto, CA 94304, USA

**Keywords:** polymer nanoparticles, nano-vaccines, immune cells, immunotherapy, tumor microenvironments, drug delivery

## Abstract

Engineering polymeric nanoparticles for their shape, size, surface chemistry, and functionalization using various targeting molecules has shown improved biomedical applications for nanoparticles. Polymeric nanoparticles have created tremendous therapeutic platforms, particularly applications related to chemo- and immunotherapies in cancer. Recently advancements in immunotherapies have broadened this field in immunology and biomedical engineering, where “immunoengineering” creates solutions to target translational science. In this regard, the nanoengineering field has offered the various techniques necessary to manufacture and assemble multifunctional polymeric nanomaterial systems. These include nanoparticles functionalized using antibodies, small molecule ligands, targeted peptides, proteins, and other novel agents that trigger and encourage biological systems to accept the engineered materials as immune enhancers or as vaccines to elevate therapeutic functions. Strategies to engineer polymeric nanoparticles with therapeutic and targeting molecules can provide solutions for developing immune vaccines via maintaining the receptor storage in T- and B cells. Furthermore, cancer immunotherapy using polymeric nanomaterials can serve as a gold standard approach for treating primary and metastasized tumors. The current status of the limited availability of immuno-therapeutic drugs highlights the importance of polymeric nanomaterial platforms to improve the outcomes via delivering anticancer agents at localized sites, thereby enhancing the host immune response in cancer therapy. This review mainly focuses on the potential scientific enhancements and recent developments in cancer immunotherapies by explicitly discussing the role of polymeric nanocarriers as nano-vaccines. We also briefly discuss the role of multifunctional nanomaterials for their therapeutic impacts on translational clinical applications.

## 1. Introduction

In managing human diseases, the biological forms of vaccines play a prominent role in activating the host’s adaptive and innate immune responses. Vaccines are commonly used as a prophylactic treatment for infectious diseases and have recently been used as therapeutic agents for cancer [1,2,3,4]. Recent developments in cancer nano-vaccines and their prime role on host immune systems have generated interest among chemical and biomedical engineers in producing novel targeted nanomedicine-based vaccines for priming the body’s immune defense to elicit anti-tumor responses [5,6,7,8]. For prospective applications, nanoparticulate adjuvants such as polymeric nanoparticles have been shown as promising antigen delivery vehicles for site-specific, targeted vaccine delivery [9]. In combination with therapeutic adjuvants, targeting agents, and immuno-therapeutic molecules, polymeric nanoparticles become potential functional candidates for therapies [10,11,12,13].

Cancer is one of the deadliest diseases and kills millions of people every year across the globe. According to the Global Cancer Observatory (GCO), an interactive web-based platform, approximately 10 million cancer deaths and 19.3 million new cancer cases were expected globally in 2020 [14]. There are a variety of cancer therapeutic approaches that exist. Among these, chemo-, photo- (thermal, dynamic), radio-, and immuno-therapies are the most evolved to target tumor cells at their sites and inducing cell death [15,16,17]. Despite good signs of progress in cancer therapeutic approaches, drug resistance, recurrence, low therapeutic responsiveness, and non-specific toxicity with undesired side effects are major concerns noted in current cancer therapies [18,19,20]. The advent and success of immuno-therapeutic strategies via applying nanomaterials are promising for patients by extending their life span. Immuno-therapeutic approaches have advantages in reducing the overall burden of primary tumors and metastatic lesions [21]. Currently, clinicians consider immunotherapy as a gold-standard treatment choice for many cancer types [22,23]. Hence, the development of various immuno-therapeutic nanoengineered antibodies and therapeutic molecules is the current focus of research for successful treatments [24].

**Figure 1:**
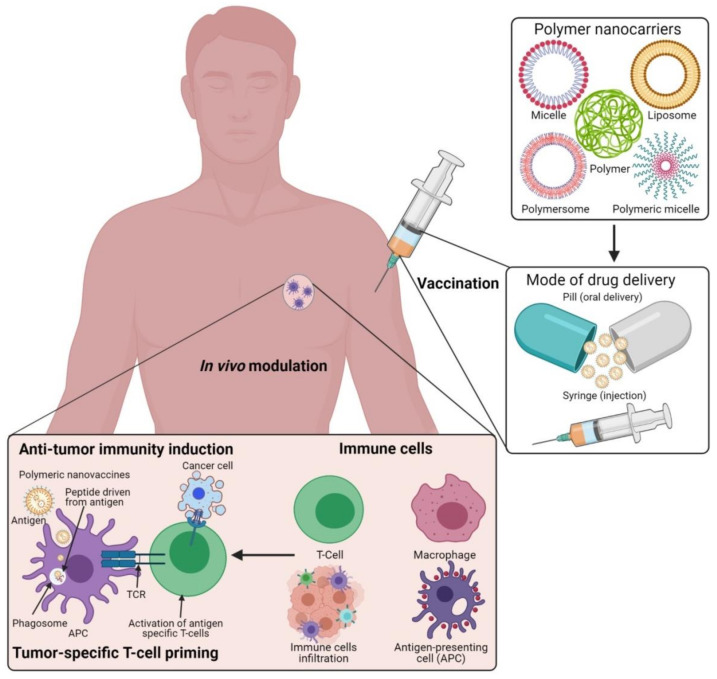
Schematic illustration of the polymeric nanocarrier systems as adjuvants for cancer vaccine deliveries and triggering host-immune-response-mediated cell death in vivo. (Images were created with the help of BioRender.com, accessed on 15 August 2021).

Currently, engineered antibodies are used to induce immune stimulation via blocking the immune checkpoints to show anticancer responses. The use of antibodies in combination with selective delivery approaches using various polymeric nanomaterials with functionalized nanocarrier systems would be a beneficial approach, and this could profoundly increase the success of treatment towards clinical applications (Figure 1). The multifunctional immunomodulatory role of nanomedicine in cancer immunotherapy is subsidizing the field of nanomaterials in immunology. Thus, the innovative developments of nanomedicine and immunotherapy through combined nanomaterial systems can enhance immunomodulatory functions in the subsequent stages of polymeric vaccine manipulations for clinical use. Occasionally, autoimmune diseases can show some unpredicted side effects in cancer immunotherapy. Polymeric nanomaterial-based cancer immuno-therapeutic strategies are shown to be more effective against hematological malignancies than solid tumors. This is mainly due to their low effectiveness in tumor penetration through their abnormal extracellular matrices, endothelial barriers, and distinct tumor microenvironments (TMEs) [25,26]. By developing highly effective polymeric nanomaterials with precise targeting of TMEs across the endothelial border, it is possible to improve the efficacy of cancer immunotherapies in solid tumors.

In recent years, engineered polymeric nanoparticles have been extensively used as a novel drug delivery platform for achieving safe and effective cancer immunotherapy [8,23,27]. The tumor immune responses and long-lasting tumor suppression effects can be achieved via vaccines like immuno-therapeutic agents, which have attracted clinicians to utilize them for their beneficial effects [28,29]. The target-specific anti-tumor immune functions of the polymeric nanoparticles can also be improved via introducing aromatic amino acid mutations in genetically encoded regions of the tumor target-specific neoantigens (non-self-peptides) [26,30]. Thus, the polymeric nanocarriers with engineered cancer neoantigen-based (cancer-specific differentially expressed antigens) vaccines have been widely used for immunotherapies via reducing autoimmunity while enhancing cytotoxic T lymphocyte (CTL) responses [27,31]. Similarly, dendritic cells (DCs) that activate antigen targeting and ligand presentation to conquer neoantigen-dependent cancer vaccines have been used to improve therapeutic functions [32,33]. It has been proven that DCs and immune regulators of type-I interferons (e.g., IFN-γ) are predominant molecules that link the innate and adaptive immunity in patients for the uptake and exposure of naive T-cells in the activation of tumor-specific CTL responses [34,35]. Several reports in cancer nanomedicine-based drug delivery approaches have revealed the functions of polymeric nanoparticles in the activation of stimulator of interferon genes (STING) and toll-like receptors (TLRs), thereby enhancing the CD8+ T-cells in preclinical animal models via stimulating the STING mechanistic pathways [36,37,38,39,40]. In this review, we elaborate on the role of engineered polymeric nanoparticles in multimodal therapeutic approaches for drug-based immuno-therapeutics in cancer. The importance of biomaterials as nanovehicles in cancer immunotherapy has been widely applied in delivering multi-targeting anticancer agents to tumors through a controlled strategy. The accomplishments of cancer immunotherapy can be achieved via implementing the following factors: (1) the use of agents that can present cancer-specific antigens to immune stimulatory cells such as antigen-presenting cells (APCs) and DCs, (2) the delivery of anticancer agents such as drug molecules and adjuvants to enhance immune responses, and (3) the use of biomolecules to modulate TMEs and improve their immune responses to anticancer immunotherapies. Hence, with the use of these nanoengineered biomaterial systems, we can potentially induce the anticancer immune responses in a variety of cancers [41]. Finally, we discuss the capabilities of polymeric nanoparticles and their clinical status as immune modifiers and possibly as therapeutic candidates in cancer therapy [42].

## 2. Evolution of Polymeric Nanomaterials and Their Engineering Strategies in Cancer Immunotherapies

Many clinical pathologies have improved immuno-therapeutic applications by the use of polymeric nanomaterials. However, understanding the mechanisms behind cancer immunity is an urgent need for deciding on suitable strategies for cancer immunotherapies [43,44]. In recent years, advancements in polymer science and nanotechnology have provided approaches to improving cancer immunotherapies. Multidisciplinary fields bridging chemistry, physics, biology, engineering, and medicine have helped design, synthesize, and characterize nanomaterials and extend their applications in various immunotherapies. Upon considering this, the biomedical investigations and the rapid developments of polymer-based nanoparticles and their deep understanding in medical fields have resulted in their clinical applications, including in cancer treatment. The potential of polymeric nanoparticles is extensive due to their unique and multifaceted functions, including adjustable surface area, easy tunability for cell-materials, and cell–chemical–biological interactions to accommodate higher drug contents during drug delivery applications [45]. The prominent phenomenon of polymeric nanoparticles is the easy surface functionalization for achieving precise target-tissue biodistribution and blood clearance upon circulation [46]. In addition, polymeric nanoparticles could also exhibit different functionalities via the incorporation of theranostic molecules such as fluorescent probes, drug conjugates, or antibody–drug conjugates. These molecules can be easily coupled onto the surfaces of the polymeric biomaterials [47,48].

The use of polymeric nanoparticles combined with metal nanoparticles to deliver cancer immunotherapies has already been approved for clinical applications and many are currently undergoing clinical trials [49]. The organic and inorganic nano biomaterials possess higher biocompatibility with reduced side effects when modified with bio-functional polymers. In addition, several organic nanoparticles with polymeric modifications have been under investigation for clinical applications as vaccines, as a means to prolong drug delivery systems, and as biocompatible dermal and tropical drugs. Likewise, the intravenous delivery of organic nanomaterials is also efficiently utilized for cancer immuno- and gene therapies [50,51]. Organic nanomaterials with the modifications of polymer materials, especially for immunotherapy, have shown broader and more prolonged success rates in preclinical models because of their increased biocompatibility with targeting efficiency [52]. Despite these, the biologically engineered polymer nanomaterials modified with organic polymers have demonstrated long-term safety with less accumulation in clearance organs such as the kidney, liver, and spleen. Hence, the utilization of polymer-based engineering approaches to make multifunctional nanoparticles has evolved to show promising delivery vehicles with increased drug loading capacity, controlled delivery, antibody targeting, and targeted accumulation at the localized TMEs as switchable candidates during therapeutic applications [53]. Upon considering all these factors, designing a polymer nanoparticle-based vaccine will always require an antigen as a primary vital content that is generally present in a short-peptide form to trigger the adaptive host immune responses [54]. Moreover, a long-lasting and robust immune response could also be achieved through natural killer (NK) cells [55]. Thus, developing the polymeric vaccine as a potential cancer immuno-therapeutic agent to effectively internalize via tumor homing and penetration through cell membranes is of immediate need for expanding this field in cancer immunotherapies.

## 3. Functional Immunotherapies Delivered to Cancer Cells via Polymeric Nanoparticles

**Figure 2:**
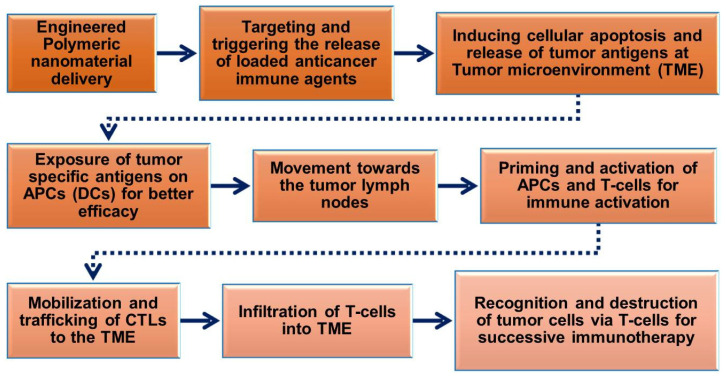
Schematic flow chart showing the roles of engineered polymeric nanomaterials in the activation and regulation of cancer immunotherapy via modulation of tumor microenvironments (TMEs).

The prerequisite of polymeric nanoparticles in cancer immunotherapy is to induce many signaling events of cancer cells to achieve treatment outcomes [56,57]. The cancer immunity cycle (Figure 2) plays a significant role in activating immunotherapy responses in the tumor to remove cancer cells or alter the TME by releasing tumor antigens. Upon delivery of polymeric nanoparticles to the tumor site, the cancer cells undergo numerous biological changes to induce apoptosis-mediated cell death pathways and release cancer antigens for further immune activation to induce CTL responses. The released cancer cancer-specific antigens are presented by the antigen-presenting cells (APCs) in the context of the major histocompatibility complexes (MHCs) [58]. MHCs play a prime role in the immune system by enhancing the modulatory functions in the infected cells during vaccination. MHCs have a highly pronounced cell surface display of intracellularly derived proteins and peptides for immune activation. Furthermore, the MHCs presenting dendritic cells (DCs) and tumor antigens stimulate the activation of immature T-cells in the lymph node to trigger the tumor-specific CTLs and natural killer (NK) cells which allow them to control cellular events and identify TMEs in tumorigenesis [32,59,60]. CTLs effectively communicate with the T-cell signaling molecules of the receptors and MHCs to attract tumor cells.

**Figure 3:**
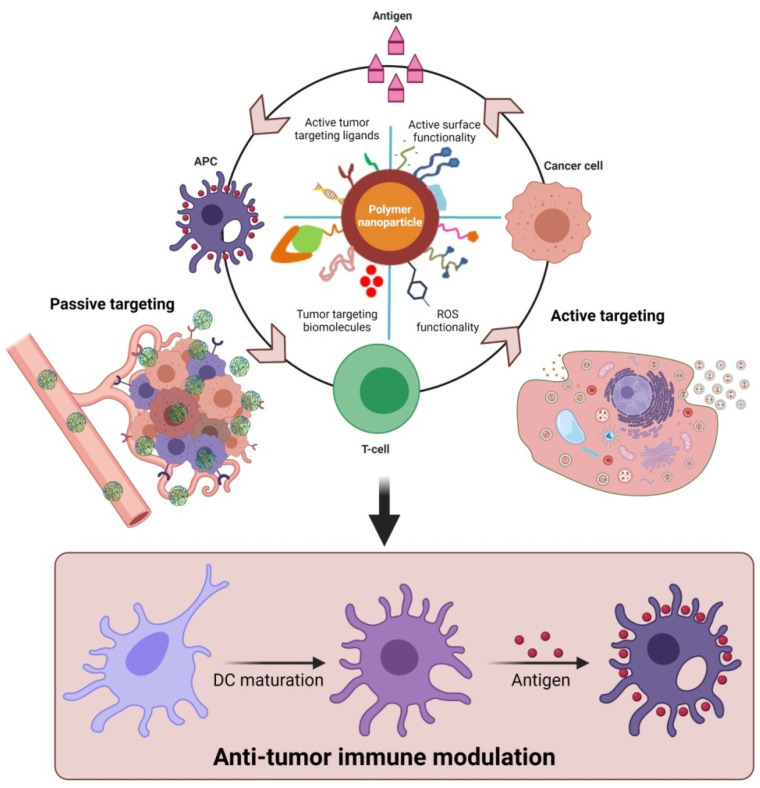
Proposed role of functional polymeric nanomaterials with immunostimulatory molecules in regulating anti-tumor potential upon delivery as cancer-specific vaccine systems. The delivery of polymer nanomaterials could enhance the tumor-specific immune modulation and improve anti-cancer responses via activation of DCs. (Images were created with the help of BioRender.com, accessed on 15 August 2021).

Furthermore, polymeric nanoparticles facilitate T-cell-mediated cellular apoptosis, which eventually stimulates the release of cancer antigens to boost further immune responses [45,50,60]. This could maintain the events that promote specific immunity to cancer, which can also be disturbed through multiple barriers that reduce the immuno-therapeutic mechanisms. In addition, the pro-inflammatory responses from the polarized macrophages (M1) [61] are highly involved in regulating and killing tumor cells via the production of immunosuppressive cytokines [62]. As shown in Figure 3, the various forms of delivered polymeric nanomaterials can facilitate the internalization of APCs through modulating migration, uptake, and maturation in the weakly acidic TMEs. This could further enable the production of tumor antigens that enhance NPs’ immunomodulatory potential, such as through reducing regulatory T-cell population, DC activation, and macrophage repolarization (M2 to M1) to induce immunogenic cell death (ICD). These actions are potentiated in the TMEs through the regulation of various cytotoxic T-cell-associated immune modulations and encouraging immunotherapies (Figure 2 and Figure 3). Immunosuppressive candidates such as PD-1, PD-L1, and CTLA-4 are involved in decreasing tumor cell proliferation. These conditions may be involved in reducing the immuno-therapeutic efficiencies that are the cause of the existing problems with current cancer immunotherapies [63,64]. Nanomaterials, including polymeric nanoparticles could intervene in these conditions, to boost cancer immunity towards successive immuno-therapeutics [65,66].

## 4. Multifunctional Nanoparticulate Systems for Cancer Immunotherapies

Multifunctional polymeric nanoparticles have been developed for various biomedical applications, specifically for the clinical diagnosis and therapies of complicated diseases like cancer. In recent years, the clinical translations of various nanoparticle systems such as mesoporous silica (MSN) [67,68], magnetic nanoparticles (MNPs) [69,70], ceria nanoparticles (CNPs) [71], carbon-based nanomaterials (CBNs) [72], gold nanoparticles (AuNPs), and polymeric nanoparticles (natural, synthetic, and semi-synthetic), have been extensively applied for cancer immunotherapies [73,74]. Among these, polymeric nanoparticles have gained tremendous attention for cancer immunotherapy since they are biocompatible, non-toxic, and easy to manipulate towards functional applications. Numerous polymers, such as chitosan, poly(ε-caprolactone) (PCL), poly(lactide acid) (PLA), polystyrene, and poly(lactic-*co*-glycolic acid) (PLGA) have been approved by the US Department of Food and Drug Administration (US-FDA) for their safe and efficient clinical use in biomedicine [75,76,77,78]. In addition, the biodegradability, biostability, and biocompatibility of polymeric materials make them suitable biomaterials for controlled drug delivery systems in chemotherapy and immunotherapy. These nanomaterials could be used as promising candidates for the effective targeting of cancers and to deliver the loaded molecules to the desired site with better efficacy, thereby enhancing the activation of immune systems [74,79]. The nano-sized (1–1000 nm in diameter) and deliverable form of drug conjugates of polymeric nanoparticles such as drug-loaded polymers, polymer-lipid nano-formulations, and polymeric drug antibody conjugates are currently playing prominent roles in nanomedicine-based therapeutic advancements [80,81].

Similarly, the physio-chemical features of polymeric nanoparticles such as morphological size, charge, shape, molecular weight, and rate of degradation determine their applications in anticancer immunotherapies [82,83]. These properties can trigger the delivered nanomaterials to produce functional events of cellular internalization, dynamics of nanomaterial-cell interaction, biodistribution, clearance, tumor targeting, and accumulation potential to induce and amplify the T-cell responses for the resultant activation of immunotherapies [84,85]. Additionally, the natural polymers of chitosan-coated nanoparticles systems and hyaluronic acid derivatives can serve as potential immune-related adjuvants that expose the immuno-stimulatory and immunomodulatory efficiencies via promoting multiple inflammatory signals of host cells [5,86,87,88,89,90]. In contrast to all these beneficial effects, the immuno-stimulatory and antigenic properties of polymers themselves are considered undesirable upon the utilization of polymeric nanoparticles as carriers to deliver antigens and adjuvants [91].

Immunotherapy for cancer using polymeric nanoparticulate systems has majorly been driven by a vaccine-specific strategic approach where tumor-cell-related substances are used to fight against cancer [43,92]. The polymeric nanoparticles carrying cancer-cell-inhibiting substances and drugs delivered to the tumor sites induce apoptosis and prevent cancer recurrence by boosting the host immune system [93]. Currently, there are several vaccines which have been developed to treat diseases associated with viral pathogens, such as human papillomavirus (HPV) and hepatitis B virus (HBV) [94,95]. In addition, there has been a wide range of cancer immuno-therapeutic vaccines that are currently at different stages of clinical trial [96,97]. The cancer vaccines are developed to induce anti-tumor immunogenicity in patients by targeting antigens differentially expressed at various stages of tumors. However, the existing problems of these vaccines are the prolonged toxicity in patients [98]. To overcome this issue, several polymeric nanoparticle-based immune-stimulating cancer vaccines with minimal non-specific toxicities are under clinical trial [99], and some of them are approved for therapeutic applications [100]. Depending on the use of polymeric vehicles, the cancer-targeting vaccines are encoded or loaded with multiple forms of tumor-homing peptides, proteins, active cellular lysates, or antigen-pulsed dendritic cells (DCs). The cancer-specific vaccines are commonly programmed to enhance or generate anti-tumor immunity via encoding suitable antigenic adjuvants [101]. These antigens could stimulate the maturation of DCs via producing danger/death signals when they encode an antigen-presenting molecule with cancer-cell properties [102]. The DC antigens could be triggered by promoting cancer vaccines to instruct the immature T-cells to promote cell-mediated cytotoxicity, thereby inducing immunological aspects of tumor clearance. The cancer vaccines designed to produce antigenic immunity to the host cells for the potential elimination of cancer cells [103]. However, antigens delivered in various forms such as tumor-associated antigens (TAAs) and tumor-specific antigens (TSAs) are extensively used to make tumor vaccines to humans as personalized therapies [92]. Thus, to develop and focus on research towards the formulation of various polymeric materials associated with therapeutic cancer vaccines, it is essential to enhance the necessary immunity by utilizing cancer-derived neoantigens and therapeutic adjuvants [104].

**Figure 4:**
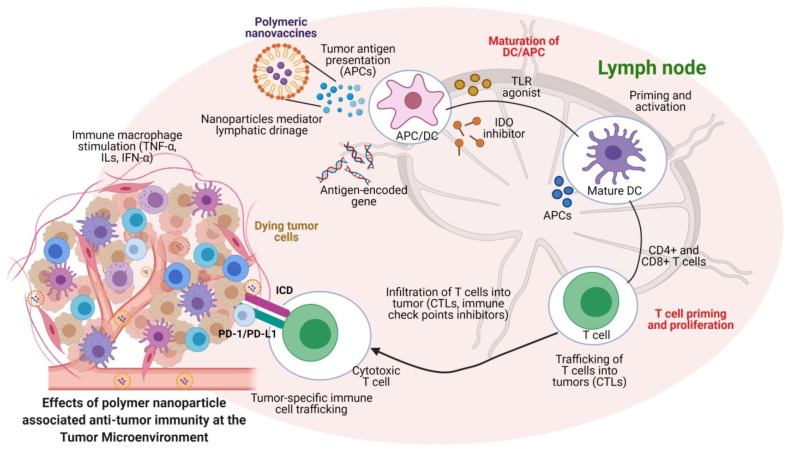
Polymeric nanoparticles regulate the lymphatic system to activate DCs to facilitate CD8+ T-cells in the tumor microenvironments (TMEs) to enhance the anti-tumor immune response. The activation of immune-related cytokines regulates various cell-death-associated functions of dying tumor cells by capturing the delivered nanoparticles from TMEs, which further mediates the ICD. The T-cell-mediated immune activation triggers the cancer cell apoptosis in TMEs. (Images were created with the help of BioRender.com, accessed on 15 August 2021).

The role of polymeric nanoparticles in immune-related events has been investigated to regulate several immunogenic signals via immune checkpoint therapies for balancing the immune system and cellular homeostasis. The commonly used immune checkpoint inhibitors in cancer therapies are designed for inhibiting receptors (e.g., programmed cell death-1 (PD-1) receptor) typically expressed in T-cells and the ligand (e.g., PD-L1) expressed by cancer cells [105,106,107]. During immune signaling, the PD-1 of the T-cell would be attracted by the cancer cells, which results in the inactivation of T-cells while promoting the tumor-associated cytokine secretion to down-regulate cancer-relevant immune responses and tumor growth [108]. A variety of polymeric nanomaterials were developed to deliver site-specific immune checkpoint inhibitors, which could also be used for the simultaneous enhancement of the potent immune checkpoint inhibition therapy [109]. Immune checkpoint inhibition therapies for cancer using biomaterials are now proven for their anticancer potential via modulation of the host cell immune mechanisms. They are also established as an alternative to conventional cancer therapies. As shown in Figure 4, the prospects of polymer nanoparticle-based drug delivery approaches for immune blockade therapies (ICB) are well demonstrated for their role in releasing agents to lymph nodes, and to the TMEs to achieve cancer immunotherapies. This suggests that polymeric nanomedicine approaches involving the use of various functionalized antibodies for immune-modulatory functions can serve as the agents to activate APCs/DCs for the improvement of ICB therapies via regulation of immune checkpoint molecules and tumor cell death associated cytokines at TMEs (Figure 4). The success of immune checkpoint inhibitor-based cancer immuno-therapeutics, such as Pembrolizumab, Nivolumab, Ipilimumab, Rituximab, Trastuzumab, Alemtuzumab, Ibritomomab, Tositumomab, Cetuximab, Bevacizumab, BMS-986016 (BMS-ONO) and GSK2831781 (mAbs) [110], DB36, DB71, DB15, and CVN (small molecules) are promising. The extension of their use to multiple applications by combining numerous polymeric materials for tumor inhibition is currently under investigation [111,112,113]. In addition, some of the side effects are being reported. These adverse effects could be easily eliminated and controlled by modifying the nature of the polymeric materials or reducing the administered doses. Concurrently, some of the patients in the clinical treatments have shown no/inadequate response to immune checkpoint inhibitor therapies. These issues can be overcome by modifying the conventional agents using polymeric substances or by changing the delivery routes, or also by combining multiple checkpoint inhibitors as combination therapies [114,115,116].

Gubin et al. [117] demonstrated the anti-PD1-1/PD-L1 and anti-CTLA-4-mediated immune checkpoint therapy for their dramatic clinical outcomes in multiple forms of cancers. Both PD-1 and PD-L1 showed prominent roles in balancing the immune homeostasis of tumors. As proven in the previous reports, immune checkpoints in tumor formation and functional T-cell differentiation into exhausted T-cells are generally known factors in inducing the immune escape and surveillance during the later stages of cancer [118]. Thus, developing novel polymeric nanoparticles using immuno-therapeutic materials could release the immune checkpoint inhibitors (ICIs) in the tumor microenvironments and improve therapeutic outcomes. This could also overcome the inefficiency and vulnerability of delivered ICIs by degradation under physiological conditions. Hence, encapsulation of ICIs by polymeric nanomaterials is emerging as a potential candidate for improving ICIs towards immunotherapy for cancers [119]. As stated in previous sections, polymeric nanoparticles can achieve and stabilize the delivered cargo to enhance the controlled release functionalities with improved therapeutic effects for longer times [120]. Moreover, studies have shown that the local delivery of immunomodulatory antibodies with sustained-release using polymeric nanoparticle systems has a promising immunotherapy potential [57,121]. In recent years, increasing immunotherapy research by applying polymer nanoparticle systems has improved the accumulation and retention of ICIs, and enhanced the related antibodies development to target tumor tissues and immune cells.

## 5. Antigen-Delivering Strategies Using Polymeric Nanomaterials to Ensure the Immuno-Therapeutic Values

**Figure 5:**
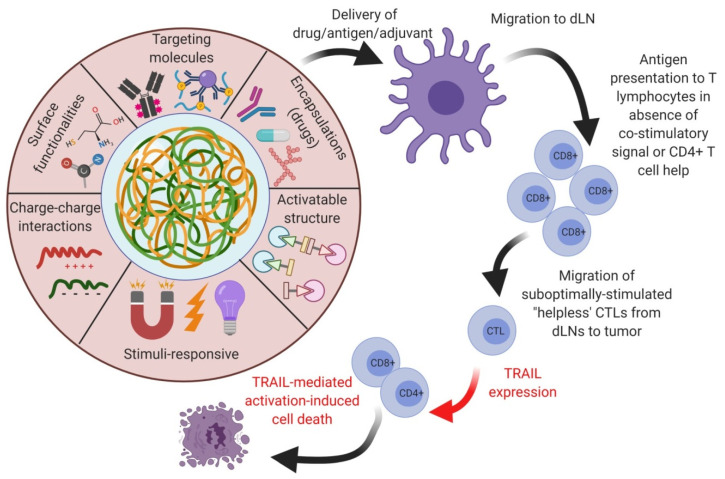
Strategic deliverable approaches of the polymeric nanomaterials as nano-vaccines to activate and regulate immune-related enhancement at the TMEs for enhanced TRAIL-mediated cellular apoptosis. (Images were created with the help of BioRender.com, accessed on 15 August 2021).

In cancer immunotherapies utilizing vaccine strategies, the DCs and macrophages play a prominent role in acting as potent vaccine-carrying vehicles. At the same time, APCs are involved in activating the immune system of host cells for both innate and adaptive immunities [122]. During this process, MHCs carry the vaccine antigens to present to APCs to extend T-cell immunity enhancements via altering their quality [123]. APCs are important immune candidates involved in the activation and presentation of tumor-specific antigens to T-cells. Hence, the application of polymeric nanomaterials to achieve nanomedicine-based anti-tumoral immune enhancements would offer novel strategies to control and administer the delivery routes, and pave the combinatorial approach to enhance the tumor-related abscopal effects by synthetic vaccines in clinical therapies. Various forms of polymeric nanoparticle delivery to the host tumor for achieving optimal efficacy in clinical settings for enhancing T-cell activation have been reported. Because of the properties of the delivered polymeric nanomaterials and the loaded antigenic peptides/targeting moieties or other associated stimuli (light and magnetic), ionic charges, and surface functionalizations or coatings could modulate the tumor-necrosis-factor-related apoptosis-inducing ligand (TRAIL) expression in TMEs for enhanced stimulation of CD8+ T-cells. This could further elucidate the TRAIL-mediated apoptotic cell death of cancers. These polymeric nanoparticles could lead to nanomedicines and vaccinations for enhanced synergistic cancer therapies (Figure 5).

Current clinical treatments involving neoantigen-based immuno-therapeutics combined with nanomaterials are inefficient due to their encoded peptides against CD8+ T-cell-mediated immune responses [123,124,125]. In contrast, gene-encoded polymer nanosystems delivered by targeted peptide-based deliverable vehicles have shown enhanced anti-tumoral immunogenicity and eliminated barriers such as limited cellular uptake and less antigen presentation via APCs and DCs [75,126]. Polymer nanoparticles and related delivery strategies of liposomes and inorganic biomaterials can also be used to overcome these issues. Moreover, targeting and controlling the release kinetics of nanoparticulate materials would also be essential to lead the high therapeutic efficacy, with improved tumor-targeting potentials to eliminate unnecessary immune responses. Hence, designing polymeric nanomaterials to control the therapeutic possibilities at the external stimuli-based target microenvironment for the loaded immuno-therapeutic molecules can reduce the non-specific toxicity to non-cancerous tissues [127,128]. Previously, liposome-based delivery of immuno-therapeutic molecules and antigen release has shown the possibility of improving therapeutic efficiency via a pH-sensitive nanoparticulate system. The delivery of antigens in the cytoplasm of DCs could facilitate an antigen-related anti-tumoral immunity to achieve immunotherapies [129,130]. Considering these approaches, the emergence of nanoparticle combined vaccine strategies for the inner and outer antigen-specific delivery could also produce a better immune response to the delivered antigens [108,131]. A list of the recently developed polymer-based nanoparticulate systems combined with therapeutic biomolecules is summarized in Table 1. Combining therapeutic molecules and imaging agents in nanocarriers can exert theranostic potential for real-time monitoring of therapeutic deliveries [116,132]. Thus, analyzing the limited demerits of the polymeric nanomaterial-based combined immuno-therapeutic systems makes it possible to achieve enormous possibilities for cellular biocompatibility and tracking of controllable delivery systems for the loaded antigens.

The currently available strategies to achieve the classical approach to cancer immuno-therapeutics via the administration of multiple antigens have failed in the clinical settings [153,154]. The therapeutic abilities of the delivered agents are primarily dependent on the individual patient’s immune system and the tumor heterogeneity. To surmount this and boost the value of immunotherapy, researchers are currently focusing on combining radiotherapy with immuno-therapeutics to achieve abscopal effects in cancer cells [15,144,155,156]. This can be highly applicable to treatment of metastatic tumors, and their control, via irradiating the primary tumors and dependent immune system enhancements can treat metastatic tumors. It has been reported that when the primary tumor is irradiated, the dying tumor cells can attract the immune cells via releasing several cell-related cytokines and damage-associated molecular patterns (DAMPs) to focus on the systemic metastatic tumors [157]. As a result, the non-irradiated metastatic tumors at distant locations could be eliminated by infiltrating the CD8+ T-cells from the primary tumor site to the metastatic TMEs. To improve this in clinical conditions, several engineered nanomaterials, including polymer-based nanomaterials, have been developed and utilized to facilitate effective interaction with the tumor cells, thereby elucidating the radiotherapeutic values towards achieving the abscopal effects [158]. Several reports using antigen-functionalized poly(lactic-*co*-glycolic acid) (PLGA) nanoparticles have been adopted to deliver the tumor-derived antigen and drugs during immunotherapy-related radiation treatments. Collectively, the polymeric nanoparticles combined with radiotherapeutic molecules enhance the significant level of production of CD8+ and CD4+ T-cells of neoantigens [108,156,159,160]. Combining polymeric nanocarriers with radiotherapy could effectively promote the tumor-associated abscopal effects at the metastatic tumor via improving the immuno-therapeutic potentials leading to anti-tumor immune response, thereby increasing patient survival [15,157]. Therefore, the value of engineered polymer nanomaterials in cancer immunotherapy can be enhanced via improving the multifunctional nature of polymer nanoparticles for cancer-specific neoantigen presentation to APCs to facilitate anti-tumor-immunity-based abscopal effects to achieve successful cancer immunotherapy in combination with radiotherapy.

In recent years, the advanced research of photodynamic therapy (PDT) or photothermal treatment (PTT) has been used for exerting immunotherapy-related abscopal effects [145,161]. In this approach, the chemotherapeutic agents with controllable nanoscale polymer materials have been used to accumulate at the tumor sites effectively. These accumulated particles generate thermal enhancement upon light-activation while releasing reactive oxygen species (ROS) at the tumor microenvironments and killing cancer cells via induction of tumor necrosis or related apoptosis. This process also releases the tumor-associated antigen that activates the immune system to display the abscopal anti-tumor effect at the metastatic sites [162]. Similarly, some chemotherapeutic agents combined with radiotherapy have effectively decreased the cancer cell growth and the release of tumor-specific antigens leading to the cancer vaccination and induction of abscopal effects [163,164,165]. These strategies would also be possible with polymeric nanoparticle systems where PD-1-PDL1 blockade therapy can kill primary and distant tumors, while biomaterials-mediated drug delivery can enhance cancer cell death through other mechanisms to achieve synergistic effects with tumor-specific immune responses and abscopal effects [166].

Additionally, the tumor-site-specific immunosuppression or the tolerance of the primary tumor to the delivered materials could affect the abscopal effects [167]. The delivery of combined chemotherapeutics and radiotherapy molecules has shown systemic toxicity in animals and has produced immune-related side effects [168]. To prevent this situation, there is an urgent need to develop biomaterial-based approaches with novel bioengineering strategies suitable for utilizing the polymeric nanoparticles to exert enormous positive feedback in cancer immuno-therapeutic conditions. As the need for immuno-therapeutic modulations via focusing the APCs of DCs and macrophages has been well proven to boost the tumor antigen presentation and phagocytosis, several types of polymeric nanoparticles have been investigated to target the human epidermal growth factor receptor 2 (HER2) and ecto-Calreticulin (ectoCRT) [169,170]. Targeting ectoCRT-based immuno-therapeutic mechanisms is a primitive form of cancer immunotherapy in CRT-mediated signaling of phagocytosis. Since several reports have explained that the “eat me” signal is a primitive form of DAMPs in immunotherapy from the dying cell surface that interacts with several phagocytic regulators to produce ICD [171,172]. The combination of chemotherapeutic molecules with the presentation of tumor antigens enhanced the levels of ectoCRT exposure to the cancer cells, followed by the tumor adaptive cancer immunity [26,173]. These CRT-based nanomedicine strategies ensure that phagocytosis is a clinically viable approach when combined with multiple forms of therapeutic molecules to enhance immunogenic cell death in cancer [174]. The polymeric nanomaterial targets the CD47 blockade of cancer cells and can impede the “don’t eat me” signaling and improve anti-tumor response. The immune-related anti-tumor responses could be achieved through the combination of therapeutic molecules; thereby, the adaptive immunity can be amplified [116,174,175]. The possible value of phagocytosis in cancer immunotherapy related to CRT might be a beneficial treatment option for consideration. It can be combined with polymer-based nanoparticles to improve tumor antigen presentation by MHCs in multiple cancer types.

## 6. Engineered Polymeric Nanomaterials to Target and Modulate Tumor Microenvironments (TMEs)

Tumor cells produce multiple cytokines, which create a favorable condition for the rapid proliferation of cancer cells. Likewise, they also make a highly immunosuppressive microenvironment that plays a critical role in restricting the immune-response-mediated cancer cell eradication, such as controlling the complex extracellular matrix (ECM), maintaining tumor hypoxia, regulating interstitial pressure in tumor tissues, creating abnormal tumor vasculature, preventing immune cell infiltration, and suppressing the CTL functions, and thereby, formation of anticancer drug resistance [11,176]. In this regard, multiple immune-related cytokines, chemokines, and immune-suppressive growth factors play significant roles in resisting anticancer drugs via modulating the TME functions [177]. Accordingly, the use of a polymer-based nanoparticle strategy has been attempted to reestablish and ensure the immune-boosting using immunoregulatory molecules to activate T-cells and immune-mediated anticancer therapies for tumor eradication [108]. Thus, engineering polymer nanomaterials to modulate the functions of tumor microenvironment-related regulatory T-cells (Tregs), tumor-associated macrophages (TAMs), and myeloid-derived suppressor cells (MDSCs) could envisage the nanomedicine strategies related to anti-tumoral immune enhancements. Hence, the recent biomaterial approaches have been focused on modulating the phases of effector T-cells contribution in anti-tumor immunity or suppressing the tumor microenvironment supportive regulatory factors for enhancement of cancer immuno-therapeutics (Figure 3 and Figure 4).

In cancer immunotherapy, the role of effector T-cells is vital in recognizing the tumor-specific antigens, maintaining cancer immunity and eradicating cancer cells [178]. Hence, the successful anti-tumoral effects could be achieved through the development of better-expressing functional molecules. Engineering functional polymer-coated or polymer-based nanomaterial encoded drugs, peptides, and targeted ligands would allow the materials to reach the cancer cells and destroy the tumors rapidly. After delivering the immuno-therapeutic agents from the nanoparticles, the agent could trigger several immunomodulatory functions with tumor-suppressive signaling to the highly active tumor microenvironments. Thus, achieving successful cancer immunotherapy with polymeric nanomaterials to restore and activate the effector cell functions via T-cell responses could moderate the tumor surface antigens in recognizing the delivered polymeric nanomaterials. Several reports have confirmed that polymer-based nanomaterials are highly involved in increasing the immune responses via improving the T-cell functions. Likewise, the effector cell modulations would play a primitive role in activating T-cell-mediated anti-tumoral immunity for the immunomodulatory and immune cell recognition processes of the pro-tumor immune cells via facilitating the tumor-microenvironment-related anti-tumor response [104,179,180]. Moreover, the immunotherapy mechanisms are highly reliable, with the effector T-cells targeting the activation process of the delivered polymeric nanomaterials. Therefore, the use of engineered polymer-based nanomaterials to modulate the anti-tumor immune rejections via delivering the antigenic peptides to MHCs would improve the effector-associated CD8+ and cytotoxic T lymphocytes (CTLs) in the tumor microenvironments (Figure 4).

## 7. Conclusion and Future Perspectives

This review collectively provided immuno-therapeutic materials based on polymeric substances to exert immune checkpoint therapy, including cancer vaccines. We have emphasized the need for polymer-based nanomedicine approaches to modulation of immunity cycles via cancer cell-specific antigen deliveries, T-cell activation, and effector T-cell function for achieving successful immunotherapy. We have highlighted the role of a diverse group of polymer nanomaterials for multiple biomedical applications, which include drug delivery, cancer therapy, and cancer immunotherapy. To improve the biocompatibility of polymeric materials, it had been widely shown that positive features have been displayed after the physio-chemical modulations, such as tunable surface complexes, increased drug packaging or loading, therapeutic encapsulation to load drugs, and the use of targeting molecules [181]. Moreover, the polymer-based nanomaterials have the unique functionality of less toxicity than conventionally available drug candidates for cancer therapy. To be specific, current progress and developments in the usage of polymeric nanomaterials in translational applications provide broader positive feedback from physicians at the clinic. In addition, their primary roles in cancer immunotherapy become convincible to achieve several immunomodulatory functions and to act as an alternative to existing conventional immuno-therapeutics. Hence, the emergence of cancer vaccines with engineered polymeric-based nanomaterials can be sufficient to replace the existing therapeutic modalities and to exert several forms of anti-tumor immune responses. Thus, materializing the polymeric nanomaterials and the motivation of engineered polymer-based nanomaterials for the next-generation cancer immunotherapy modulations could be achievable for personalized medicine in healthcare systems.

Moreover, the combinatory therapeutic values could also activate immune mechanisms at the primary tumor cells that target the secondary metastatic tumors to induce abscopal effects. The existing challenges of the conventional immunotherapies could also be overcome via polymeric-nanoparticles as targeted delivery vehicles while also improving translational application by developing personalized therapy. Overall, the field of polymer-based nanomaterials in cancer immunotherapy is still at an early stage and needs further improvements to achieve possible cancer immunotherapy-related breakthroughs in the design and development with advanced combinatorial strategies for the potential progress of personalized cancer vaccines to the clinics. However, this field is of highly interdisciplinary research with several biomedical strategies, and they are evolving to the improvable immuno-therapeutics for cancer. The potential anticancer immunotherapy that needs polymer biomaterials to interact with the immune system is a primitive one but is important for the focused growth of this research. Overall, a better understanding of this interdisciplinary research could improve patients’ lives, and could emphasize development of novel biomaterials-based cancer vaccines, especially with the use of polymeric nanomaterials for clinical applications.

## Figures and Tables

**Table 1:** A list of polymeric nanomaterials and their combinations with loaded anticancer molecules evaluated for the potential cancer immuno-therapeutic functions in vitro and in vivo.

Nanocarriers	Drugs/Biomolecules	Targeted Therapeutics	Reference
**Chitosan nanoparticles (CS NPs)**	Polyinosinic-polycytidylic acid sodium salts	Next-generation vaccines to bypass the ex vivo manipulation and induce immune responses by targeting toll-like receptor 3 (TLR3) in endosomes.	[133]
**Chitosan-conjugated copper oxide nanoparticles (CS@CuONPs)**	Specific antigen conjugated vehicle for antigen delivery	To activate macrophages and trigger CTLs to induce cell death.	[76]
**Chitosan-coated selenium nanoparticles (CS@SeNPs)**	Folic acid-functionalized CS@SeNPs targeting moiety for mRNA delivery	To act as tumor vaccine and immunotherapy with amplified immune response.	[77]
**Chitosan and gallic acid grafted chitosan nanoparticles (GACS NPs)**	Cyclophosphamide (CPA)	To exhibit significant immune stimulation in CPA-treated mice in vivo.	[134]
**Lipid nanoparticles (LNPs)**	Type A CpG oligodeoxynucleotides (ODNs)	To induce Th1 and CD8 T-cells skewed immune environment and without toxicity via activating Th1 and CD8+ T-cells-skewed immune modulation.	[135]
**Functionalized LNPs**	Oligodeoxynucleotides (ODNs) with unmethylated cytosine-phosphate-guanine (CpG) motifs (CpG ODNs)	Augmented the adjuvant vaccine effects of CpG-ODN and increased protective spectrum of conventional influenza split vaccine.	[136]
**mRNA-LNPs**	TLR agonist mono-phosphoryl lipid A (MPLA)	Induced T-cell immunity without the strong induction of type I-IFNs under the reduced DCs activated TMEs.	[137]
**Lipid-base nanoparticles (immunoliposomes)**	Toll-like receptor 7 (TLR7) agonist TMX-202	To display high specificity over lymphocytes that showed adequate TLR-specific secretion of the anti-cancer cytokines *viz* IL-12p70, INF-α 2a, and INF-γ.	[138]
**Lipid-modified DNA NPs**	Immune adjuvant CpG motifs	To show up-regulation of a co-stimulatory molecule, cytokine expression and production of the pro-inflammatory cytokine, and activation of DCs in TME.	[139]
**Modified LNPs**	Pam3 and OVA-mRNA	To exhibit expression of tumor antigens with enhanced immune stimulation via improving the tumor prevention efficacy by administered mRNA vaccines.	[140]
**Mannose-functionalized antigen nanoparticles (MAN-OVA/PEI NPs)**	Antigen ovalbumin (OVA) vaccine delivery system	To accelerate endosomal/lysosomal escape and increase MHC-I antigen presentation to B3Z T-cell hybridoma.	[29]
**Poly(lactic-*co*-glycolic acid) (PLGA NPs)**	Chemokine (C-C motif) receptor-2-shRNA (CCR2-shRNA- EGFP-EGF1) and Coumarin-6	To be effectively taken up by an atherosclerotic cellular model of macrophages and target-silence corresponding CCR2 mRNA expression for the use of developed NPs in the therapy of atherosclerosis.	[141]
**PEGylated PLGA microsphere**	Self-assembled poly, tetanus toxoid (tt)	Up-regulation of expressions of IL-6, TNF-α, IL-12p70, and IL-10 concludes the surface assembled microspheres as vaccine adjuvant.	[142]
**Combinatory PLGA NPs**	Combined with TLR7/8 bi-specific agonists	PLGA NPs triggered DC activation, which led to enhanced maturation, induction of CD8+ T-cells, and significant anti-cancer efficacy in vivo.	[125]
**Functionalized PLGA NPs**	OVA antigen, and polyinosinic-polycytidylic acid sodium salt (toll-like receptor 3; TLR3)	Achieved a vaccination with PLGA NP-treated DCs that helped generate OVA-specific CD8+ T-cells and enhanced anti-tumor efficacy via delivering tumor-specific antigen and adjuvants to DCs.	[40]
**Modified PLGA NPs**	Indocyanine green (ICG), imiquimod (R837), and toll-like receptor 7 (TLR7) agonist	Clinically approved components showed NIR light-triggered photothermal ablation, generating tumor-associated antigens, and this adjuvant showed vaccine-like functions.	[143]
**Core-shell PLGA NPs**	Imiquimod (R837) and TLR7 agonist	To significantly improve the radiotherapy efficacy by reducing the tumor hypoxia and modulating the immune-suppressive TMEs, which enables synergistic local treatments for clinical translation.	[144]
**pH-responsive nanovesicles self-assembled of block copolymer polyethylene glycol-b-cationic polypeptide (pRNVs)**	Photosensitizer 2-(1-hexayloxyethyl)-2-devinyl pyropheophorbide-a (HPPH), and indoleamine 2,3-dioxygenase inhibitor, indoximod (IND)	To serve as nanocarriers and immunogenic cell death (ICD) via pre-apoptotic exposure of calreticulin, which further enabled the photodynamic cancer therapy and increased dendritic cell (DC) recruitment and immune response provocation after ICD induction.	[145]
**Adjuvant NPs**	Imidazoquinoline moiety	Delivered NPs were effectively internalized by immature DCs and exhibited enhanced in vivo activation, facilitating multivalent interactions between delivered moieties and endosomal TLR7.	[146]
**Poly(L-histidine and hyaluronic acid) NPs**	Immune regulator 848 and doxorubicin (DOX)	Dual pH-responsive NPs exhibited enhanced tumor-targeting ability and growth inhibition via regulating anti-tumor immunity and killing cancer cells. The release of R848 and DOX achieved synergistic effects of immunotherapy and chemotherapy against breast cancer.	[147]
**Silk-fibroin nanoparticles (SF NPs)**	Doxorubicin (DOX)	Magnetic NPs and DOX-loaded SF NPs exhibited magnetic-field-induced tumor-targeting ability and effective chemotherapy of multidrug resistance and thereby imaging and drug delivery carriers in vivo.	[132]
**SF NPs**	5-fluorouracil (5-FU) and curcumin	SF NPs with drug 5-FU and curcumin showed controlled release and anti-cancer effects induced by apoptosis of cancer cells via generation of cellular reactive oxygen species in vitro.	[148]
**Carboxylated polystyrene particles**	Ovalbumin (OVA)	Shape- and size-controlled nanocarrier importance is demonstrated in modulating immune responses. This is a highly relevant physical attribute for antigen-presenting nanocarriers for immune modulation.	[149]
**Protein NPs made of polymerized OVA and chemically linked poly(ethylene) glycol (PEG)**	Antigen OVA	To show activity in dendritic cells, resulting in higher OT-I CD8+ cells proliferation in vitro. NPs enhanced lymphatic drainage in vivo and increased uptake by lymph node macrophages, dendritic cells, and B cells thereby expressed higher anti-OVA antibody titers to signify the improved humoral immune responses in vivo.	[150]
**Polylactic acid (PLA) gigaporous microsphere**	Antigens such as OVA, mucin 1 (MUC1) and neoantigen	Self-healing microsphere-based vaccine showed a potent T-cell response, combined with immune checkpoints inhibitors that facilitated improved performance in cancer vaccination.	[151]
**Matrix protein 2 ectodomain (M2e) and M2e-neuraminidase (M2e-NA) fusion protein NPs**	M2e and M2e-NA proteins	Double-layered protein NPs containing structure-stabilized M2e and NA can be utilized to develop into universal influenza virus vaccines. Moreover, layered protein NPs can be a vaccine platform for pathogens.	[152]

## Data Availability

Data sharing is not applicable.
